# Carpal Tunnel Release With Ultrasound Guidance: Intermediate-Term Clinical Outcomes and Magnetic Resonance Imaging Findings

**DOI:** 10.1016/j.jhsg.2023.05.002

**Published:** 2023-06-07

**Authors:** Grace E. Nicholas, Jen Galloway, Jennifer Hawley, Joseph C. McGinley

**Affiliations:** ∗ Department of Radiology, University of Washington School of Medicine, Seattle, WA; † The McGinley Clinic, Casper, WY

**Keywords:** Carpal tunnel syndrome, Carpal tunnel release, CTR-US, Magnetic resonance imaging, WALANT

## Abstract

**Purpose:**

The purpose of this study was to report intermediate-term outcomes following carpal tunnel release using ultrasound guidance and wide-awake local anesthesia no tourniquet, including a subset of patients with preoperative and postoperative magnetic resonance imaging (MRI).

**Methods:**

In this observational study, patients with carpal tunnel syndrome were treated with carpal tunnel release using ultrasound guidance and wide-awake local anesthesia no tourniquet in a procedure room at a single center. Main outcomes were complications; return to activity and work at 2 weeks; Quick Disabilities of the Arm, Shoulder, and Hand and Boston Carpal Tunnel Questionnaire scores through 6 months; and postoperative morphological changes of the transverse carpal ligament, median nerve, and carpal tunnel evaluated using MRI.

**Results:**

No complications were reported among 65 patients (68% women, 96 wrists). By 2 weeks, 97% of patients returned to normal activity and 100% returned to work. Statistically significant improvements in Boston Carpal Tunnel Questionnaire symptom severity scale, Boston Carpal Tunnel Questionnaire functional status scale, and Quick Disabilities of the Arm, Shoulder, and Hand scores occurred by the 2-week follow-up interval and persisted at 6 months (all *P* < .001). Pre- and postoperative MRI scans were available for 13 patients (17 wrists) at the 3-month mean follow-up. Complete transverse carpal ligament transection was documented in all wrists. Key MRI findings included a 22% increase in carpal tunnel cross-sectional area at the hamate (*P* < .001), a 52% increase in median nerve cross-sectional area at the hamate (*P* < .001), an 18% reduction in median nerve signal intensity (*P* = .002), a 38% reduction in the flattening ratio of the median nerve at the hamate (*P* < .001), a 33% reduction in the flattening ratio of the median nerve at the pisiform (*P* < .001), a 20% reduction in the flattening ratio of the carpal tunnel at the hamate (*P* < .001), and a palmar shift of the median nerve relative to the hamate in all cases.

**Conclusions:**

Carpal tunnel release using ultrasound guidance using wide-awake local anesthesia no tourniquet in a procedure room setting was safe, effective, and resulted in morphological changes that were consistent with carpal tunnel decompression as demonstrated by MRI.

**Type of study/level of evidence:**

Therapeutic IV.

Carpal tunnel syndrome (CTS) is the most common entrapment neuropathy^1^^,^^2^ and is associated with high social and economic costs.^2^ Initial treatment typically includes activity modification, physical therapy, splinting, nonsteroidal anti-inflammatory drugs, or corticosteroid injections.^3^ For patients with severe or refractory symptoms, surgical release of the transverse carpal ligament (TCL) may be indicated.

Although multiple studies have reported a successful symptomatic relief for patients following traditional open, mini-open, and endoscopic carpal tunnel release (CTR), techniques continue to evolve with a focus on reducing surgical morbidity and accelerating recovery.^4^, ^5^, ^6^, ^7^ More recently, CTR with ultrasound guidance (CTR-US) has been proposed as a technique with a limited incision size that maintains satisfactory visualization.^3^^,^^8^, ^9^, ^10^ CTR-US techniques generally use a minimally invasive approach (incision <5 mm) to transect the TCL while using real-time ultrasound guidance to continuously monitor critical anatomy during the procedure.^8^, ^9^, ^10^

Multiple studies have reported statistically significant and clinically meaningful improvements in patient-reported outcomes following CTR-US.^3^^,^^8^, ^9^, ^10^, ^11^, ^12^, ^13^, ^14^, ^15^ Despite these encouraging clinical results, the morphological changes within the carpal tunnel following CTR-US have not been fully characterized. Previous research has used magnetic resonance imaging (MRI) to document morphological changes consistent with carpal tunnel decompression (eg, TCL transection, increased carpal tunnel dimensions, and reduced median nerve compression) following open and endoscopic carpal tunnel release (ECTR).^16^, ^17^, ^18^, ^19^, ^20^ However, only one study has used MRI to evaluate post–CTR-US changes in the carpal tunnel.^21^ Although the authors documented complete TCL transection and reduced median nerve compression, they did not report additional important morphological indicators of carpal tunnel decompression such as an increased carpal tunnel cross-sectional area and reduced median nerve edema.^16^, ^17^, ^18^, ^19^, ^20^, ^21^ Consequently, the purpose of this study was to report intermediate-term outcomes following CTR-US and WALANT, including a subset of patients with pre- and post–CTR-US MRI scan including the assessment of the TCL, median nerve, and carpal tunnel dimensions.

## Materials and Methods

This study was reviewed and approved by the Wyoming Medical Center institutional review board.

### Participants

This was a retrospective observational study of prospectively collected data from patients with CTS treated with CTR-US and WALANT in a procedure room at a single center between July 2019 and October 2021. Eligible patients had moderate to severe CTS confirmed by history, physical examination findings, and electrodiagnostic testing, and they underwent baseline MRI.

### Procedures

All procedures were performed by the same physician (who had 13 years of experience in ultrasound-guided procedures) and a single assistant in a procedure room setting using a WALANT technique.^22^ Through a small incision (4–5 mm) and using ultrasound guidance with augmented reality for ultrasound (McGinley Education Innovations, LLC), the TCL was transected using a commercially available device (UltraGuideCTR, Sonex Health, Inc) designed to facilitate CTR-US by creating space in the carpal tunnel followed by TCL transection using a retrograde knife.

Key procedural steps have been previously published.^3^^,^^12^^,^^15^ A preprocedural ultrasound was performed using a high-frequency 17MHz linear transducer (Acuson Freestyle, Siemens USA) to mark the borders of the transverse and longitudinal safe zones as well as the incision site in the distal forearm. The forearm was prepared and draped in the usual sterile fashion. Using a sterile ultrasound cover and sterile gel, relevant anatomical landmarks were again identified, including but not limited to the median nerve, thenar motor branch/recurrent motor branch of the median nerve, palmar cutaneous branch of the median nerve, median and ulnar digital nerves and any communications, ulnar vessels and superficial palmar arch, and distal TCL. The safe zones were re-scanned to ensure acceptable anatomy and sonographic visualization.

A #15 scalpel blade was used to create a 4–5 mm incision at the proximal wrist crease, penetrating the antebrachial fascia. A sterile stainless-steel dilator was then passed into the transverse safe zone using direct ultrasound guidance to further free the synovial tissue from the ligament and facilitate device insertion. Direct ultrasound guidance was then used to pass the cutting device into the carpal tunnel and position it within the transverse and longitudinal safe zones. Following ultrasound confirmation of the appropriate device positioning distal to the TCL and with respect to the surrounding neurovascular structures, balloons were deployed to create space in the carpal tunnel, and the position of the device was re-assessed. Following a satisfactory re-assessment, the cutting knife was activated from its recessed position, and the TCL was transected in a retrograde manner using direct ultrasound guidance and up to three passes. The cutting knife was then placed in its proximal recessed position, the balloons were deflated, and the TCL was probed using the stainless-steel dilator to ensure complete ligament transection and release of the median nerve from the ligament and adjacent synovial tissues. Wounds were closed with sterile adhesive strips; none of the procedures required sutures. Following closure, wounds were dressed in sterile gauze and sterile film, followed by a compression wrap. Patients were instructed to avoid driving and strenuous activity for the remainder of the day and were advised to use nonsteroidal anti-inflammatories or acetaminophen and ice for pain and edema control. Opioids were not prescribed for pain control. Patients were instructed to wear a wrist brace at night for 10 days and were provided with recommended post-CTR stretches and exercises beginning on postoperative day 2 and completed as tolerated. Patients were allowed to return to activities and work as tolerated, starting the day after the procedure. Postprocedure follow-up occurred in the clinic 3 weeks after surgery.

### Outcomes

Before surgery, patients provided basic demographic information including age, sex, employment status, and job description (desk-based, repetitive light manual, or heavy manual). Return to activity (normal activities outside of work) and return to work statuses were collected at 2 weeks after surgery. Before and after surgery at 2 weeks, 1 month, 3 months, and 6 months, patients were asked to complete the Quick Disabilities of the Arm, Shoulder, and Hand (*Quick*DASH) questionnaire and the Boston Carpal Questionnaire symptom severity scale (BCTQ-SSS) and functional status scale (BCTQ-FSS). The *Quick*DASH is an 11-question survey that assesses upper limb physical symptoms and function of patients on a scale of 1 (asymptomatic/no difficulty) to 5 (extreme/unable to perform). A score is generated from 0 to 100, with higher scores reflecting more symptoms/disability.^23^ The BCTQ is a commonly used patient-reported outcome measure for CTS.^24^ The BCTQ-SSS has 11 questions and uses a five-point rating scale, and the BCTQ-FSS has eight items that are rated for a degree of difficulty on a five-point scale. Each scale generates a final score ranging from 1 to 5, with a higher score indicating a greater disability. The BCTQ has undergone extensive testing for validity, reliability, and responsiveness.^25^ Questionnaires at 1, 3, and 6 months were administered online predominantly or by phone or an email at patient request. Reminder phone calls were placed to patients who did not complete their questionnaires in a timely manner.

### Magnetic resonance imaging studies

All patients were offered preoperative and postoperative MRI. Not all patients were able to have MRI scans. Only patients with both pre- and post–CTR-US MRI scans are included in this study. All postoperative MRI measurements were measured blinded to the preoperative MRI data. Following postoperative MRI analyses, the preoperative MRI parameters were measured and comparisons were assessed. All measurements were performed by the primary author and verified by the interventional radiologist who performed all procedures for this study. T1- and T2-weighted axial, coronal, and sagittal high-resolution sequences along with volumetric acquisitions using a Siemens 1.5T and 3.0T scanner were collected. Imaging analysis followed previously published guidelines for measuring morphological changes involved in CTS.^16^, ^17^, ^18^, ^19^, ^20^, ^21^ The TCL status was reported as intact or transected. The TCL was considered to be transected if there was a discontinuity and separation throughout the proximal–distal length of the carpal tunnel on the postoperative MRI. A palmar shift of the median nerve at the level of the hook of hamate was defined as the postoperative change in the distance between the palmar aspect of the carpals and the center of the median nerve. The flattening ratio of the median nerve was measured at the level of the hook of the hamate and the level of the pisiform and was defined as the ratio of the long cross-sectional diameter to the short cross-sectional diameter, with a larger ratio representing a flatter median nerve. The flattening ratio of the carpal tunnel was similarly measured at the level of the hook of the hamate. Median nerve signal intensity was measured to assess changes in median nerve edema. Finally, the cross-sectional areas of the carpal tunnel and median nerve were both outlined digitally and measured at the level of the hamate using Terarecon 3-dimensional software (Terarecon, Inc).

### Statistical analysis

Based on multiple repeated measurements of BCTQ-SSS, BCQ-FSS, and *Quick*DASH in individual participants, we used a linear mixed model with Bonferroni correction that incorporates all available data from patients with a pretreatment and at least one posttreatment observation. Data at each follow-up interval were referenced to the pretreatment data and modeled as fixed effects, with a random effect specified at the procedure level. Follow-up data were reported as the mean and 95% confidence interval. Changes in MRI parameters were analyzed with a paired samples t-test for continuous data and a Wilcoxon signed-rank test for paired categorical data. Two-sided *P* values of less than .05 were considered statistically significant.

## Results

A total of 65 patients (96 wrists) were treated consecutively with CTR-US in this study. All procedures were completed successfully using local anesthesia. No procedures were discontinued because of pain or poor visualization. Procedures were typically completed using two to three passes to cut the TCL. No complications occurred intraoperatively or during follow-up. Among the 65 patients (68% women), 34 (52%) had a unilateral procedure, 19 (29%) had simultaneous bilateral procedures, and 12 (18%) had staged bilateral procedures. The CTR-US procedure was performed on the dominant hand in 52 (80%) patients. Among the 41 (63%) employed patients, 20 (49%) reported desk-based duties, 13 (32%) reported repetitive light manual duties, and eight (20%) reported heavy manual duties.

Among the 60 (92%) patients with available time to return to normal activity data, 58 (97%) reported returning to normal activities by 2 weeks. Among the 38 (93%) employed patients with available time to return to work data, 38 (100%) reported returning to work by 2 weeks, with 34 returning to full work duties and four returning to limited duties.

**Figure 1:**
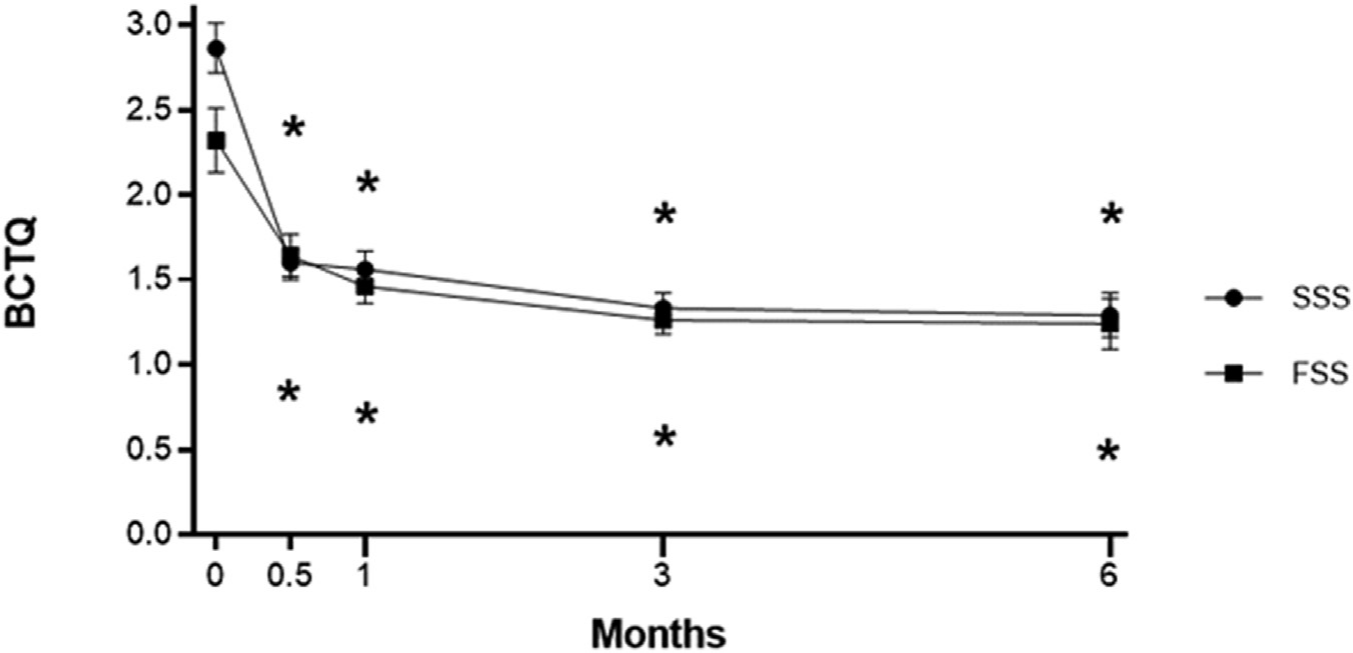
Change in BCTQ-SSS and BCTQ-FSS score over 6 months following CTR-US. Plotted data are mean and 95% confidence interval. Asterisk denotes *P* < .001 for change compared to baseline using a Bonferroni-adjusted linear mixed model.

**Figure 2:**
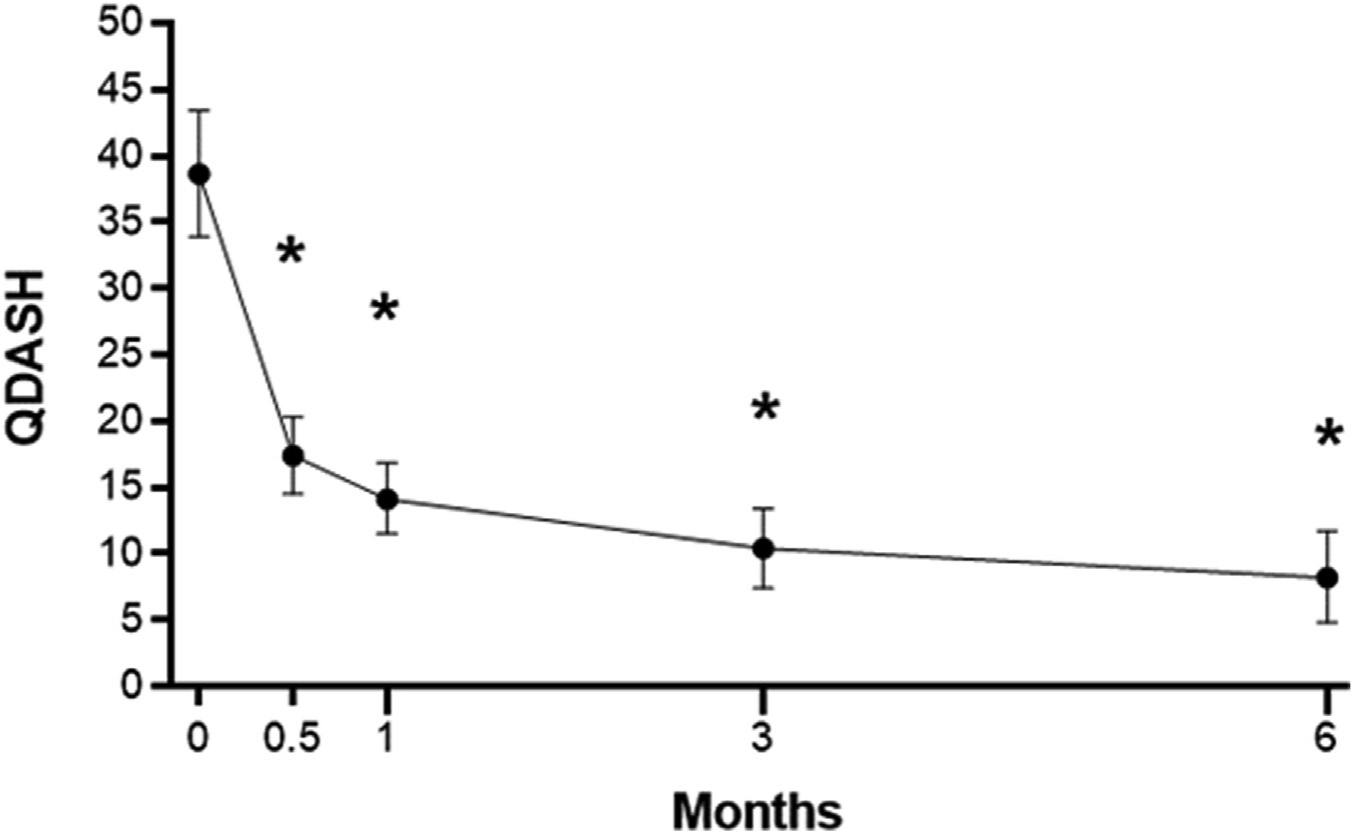
Change in *Quick*DASH score over 6 months following CTR-US. Plotted data are mean and 95% confidence interval. Asterisk denotes *P* < .001 for change compared to baseline using a Bonferroni-adjusted linear mixed model.

Patient-reported outcome data were available in 65 (100%) patients between 2 weeks and 1 month after surgery and in 39 (60%) patients between 3 and 6 months after surgery. Statistically significant improvements in BCTQ-SSS, BCTQ-FSS, and *Quick*DASH scores occurred by the 2-week follow-up interval and persisted at 6 months postprocedure (*P* < .001 for each outcome at each follow-up interval) (Table 1 and Figs. 1 and 2). The improvements exceeded the previously published minimal clinically important difference values of 15 points for *Quick*DASH,^26^ 1.17 points for BCTQ-SSS,^27^ and 0.74 points for BCTQ-FSS^27^ at each follow-up interval, with the exception of BCTQ-FSS at 2 weeks.

**Table 1:** Changes in Patient-Reported Outcomes Following CTR-US∗

Outcome	2 Wk	1 Mo	3 Mo	6 Mo
BCTQ-SSS	−1.26 (−1.46, −1.07)	−1.30 (−1.52, −1.08)	−1.53 (−1.75, −1.31)	−1.58 (−1.83, −1.33)
BCTQ-FSS	−0.68 (−0.90, −0.46)	−0.86 (−1.11, −0.61)	−1.06 (−1.32, −0.80)	−1.08 (−1.39, −0.78)
*Quick*DASH	−21.2 (−26.7, −15.8)	−24.5 (−30.8, −18.3)	−28.2 (−35.1, −21.4)	−30.4 (−37.7, −23.1)

ANOVA, analysis of variance.

∗ Values reported as mean change (95% confidence interval) from pretreatment calculated from linear mixed-model ANOVA with Bonferroni correction.

**Figure 3:**
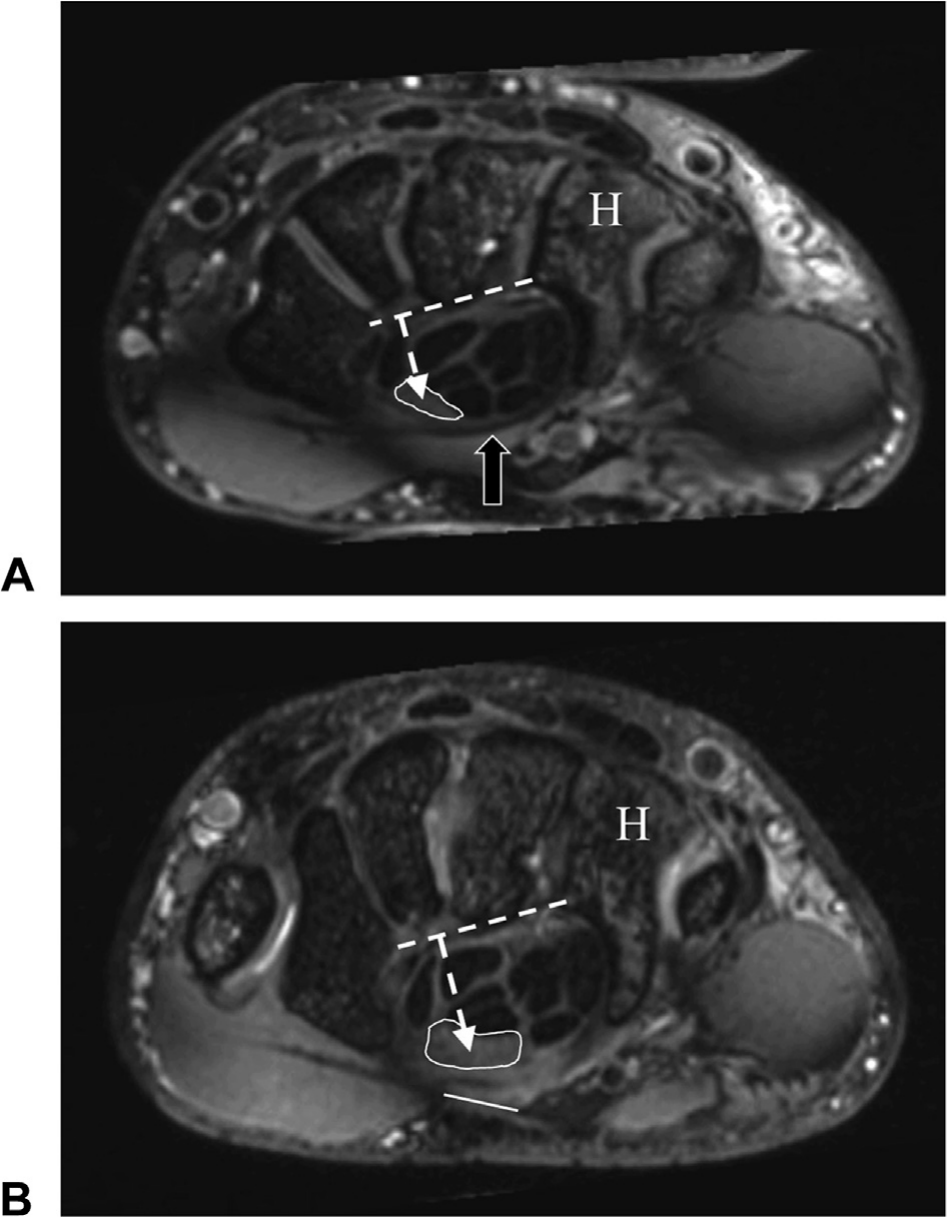
**A** Preoperative T2 weighted axial MRI at the hamate (H) level shows the cross-sectional area of median nerve (white outline) with the transverse carpal ligament intact (arrow). **B** 3-months postoperative T2 weighted axial image at the hamate level shows an increase in the cross-sectional area of median nerve (white outline) and a gap in the transected transverse carpal ligament (white line). Dashed line and arrow in images **A** and **B** show a palmar shift of median nerve reported as the difference in postoperative distance measured from the palmer aspect of the carpals (dashed line) and the center of the median nerve (dashed arrow).

**Figure 4:**
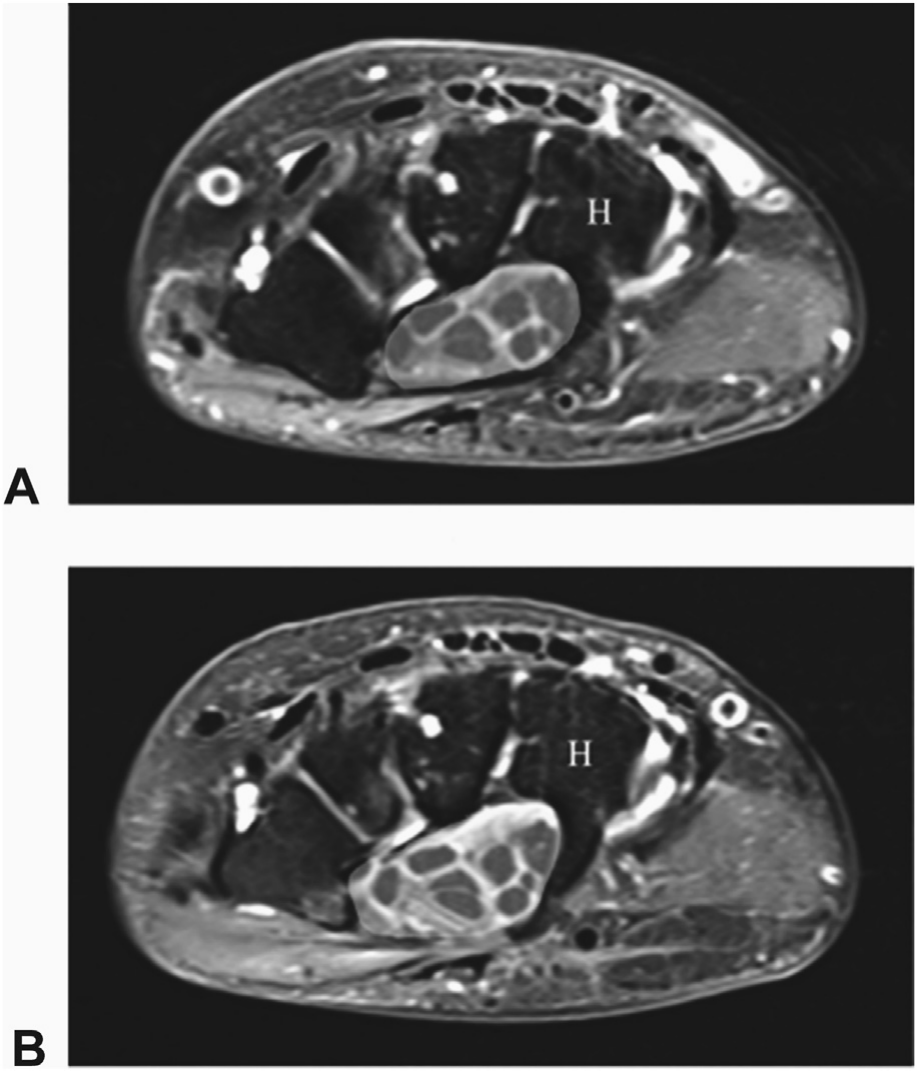
**A** Preoperative T2 weighted axial MRI at the hamate (H) level shows the cross-sectional area of carpal tunnel (yellow shading). **B** 4-months postoperative T2 weighted axial image at the hamate level shows an increase in the cross-sectional area of the carpal tunnel (yellow shading).

MRI scans were available on 13 patients/17 wrists at a mean of 3 months following surgery (range, 1–7 months). The 3-month clinical outcomes of patients who returned for an MRI were comparable with those who did not (BCTQ-SSS: 1.5 ± 0.4 vs 1.3 ± 0.3; BCTQ-FSS: 1.1 ± 0.2 vs 1.3 ± 0.3; *Quick*DASH: 7 ± 8 vs 10 ± 10), suggesting that the MRI findings are representative of the entire sample. MRI parameters are summarized in Table 2. Complete transection of the TCL was documented in all 17 wrists. Key postoperative MRI findings including a 22% increase in the cross-sectional area (CSA) of the carpal tunnel at the hamate (*P* < .001), a 52% increase in the median nerve CSA at the hamate (*P* < .001), an 18% reduction in the median nerve signal intensity (*P* = .002), a 38% reduction in the flattening ratio of the median nerve at the hamate (*P* < .001), a 33% reduction in the flattening ratio of the median nerve at the pisiform (*P* < .001), a 20% reduction in the flattening ratio of the carpal tunnel at the hamate (*P* < .001), and a palmar shift of the median nerve relative to the hamate were appreciated in all cases. These observed changes are consistent with the decompression of the carpal tunnel, reduction in median nerve compression in the tunnel (especially distally at the hamate), and reduction in intraneural edema.^16^, ^17^, ^18^, ^19^, ^20^, ^21^ Representative preoperative and postoperative MRI images are provided in Figures 3 and 4.

**Table 2:** Changes in MRI Parameters Following CTR-US

MRI Parameter	Pre∗	Post∗^,^†	Change (%)	Change (Absolute)	95% CI (Absolute)	*P* Value
Carpal tunnel, hamate level						
Width (mm)	22.0 ± 1.7	21.9 ± 2.6	0%	−0.1	−1.1, 0.9	.82
Height (mm)	11.3 ± 1.5	14.1 ± 2.0	24%	2.7	1.9, 3.6	<.001
CSA (mm^2^)	215 ± 39	263 ± 44	22%	48	34, 62	<.001
Flattening ratio‡	1.96 ± 0.25	1.57 ± 0.23	−20%	−0.39	−0.55, −0.23	<.001
Median nerve, hamate level						
Long diameter (mm)	6.73 ± 1.95	5.71 ± 2.01	−15%	−1.02	−1.77, −0.28	.01
Short diameter (mm)	2.25 ± 0.70	3.01 ± 0.95	34%	0.76	0.35, 1.17	.001
CSA (mm^2^)§	10.1 ± 6.7	15.4 ± 8.4	52%	5.3	3.8, 6.8	<.001
Flattening ratio‡	3.17 ± 0.98	1.96 ± 0.56	−38%	−1.21	−1.68, −0.75	<.001
Volar distance (mm)	9.22 ± 1.43	11.08 ± 2.10	20%	1.86	1.11, 2.61	<.001
Signal intensity	912 ± 288	749 ± 265	−18%	−163	−253, −72	.002
Median nerve, pisiform level						
Short diameter (mm)	2.84 ± 0.98	3.46 ± 1.10	22%	0.62	0.18, 1.06	.008
Long diameter (mm)	8.10 ± 1.64	6.96 ± 1.76	−14%	−1.13	−2.1, −0.17	.02
Flattening ratio‡	3.13 ± 1.06	2.10 ± 0.54	−33%	−1.03	−1.46, −0.6	<.001
Volar distance (mm)	11.8 ± 2.7	13.3 ± 2.9	13%	1.5	0.9, 2.1	<.001
Median nerve position‖						
Dorsal to line	6% (1/17)	0% (0/0)				<.001
Crosses line	94% (16/17)	6% (1/17)				
Palmar to line	0% (0/0)	94% (16/17)				

CI, confidence interval; CSA, cross-sectional area.

∗ Values reported as mean ± SD or percent (n/N).

† MRI performed at mean 3 months (range, 1–7 months) after CTR-US.

‡ Flattening ratio was defined as the ratio of the long cross-sectional diameter to the short-cross-sectional diameter, with the larger ratio representing a flatter median nerve.

§ Increase in the median nerve CSA is expected at the hamate following successful decompression.

‖ Center of the median nerve was compared to a line drawn from the hook of the hamate to the ridge of the trapezium. All nerves demonstrated a palmar shift at the time of post–CTR-US MRI.

## Discussion

The purpose of this study was to report intermediate-term outcomes following CTR-US using WALANT, including expanded post–CTR-US morphological data and a subset of patients with preoperative and postoperative MRI. Our results demonstrated that CTR-US using WALANT in a procedure room setting was safe, effective, and showed clinical improvements in validated patient-reported outcomes as early as 2 weeks after surgery, which were maintained throughout the 6-month follow-up period. These results of rapid improvement are similar to those reported in previous studies using US-guided techniques and similarly less-invasive CTR techniques such as ECTR.^3^^,^^8^^,^^10^^,^^12^^,^^15^^,^^28^^,^^29^

Several studies have reported clinical outcomes following CTR-US using *Quick*DASH and BCTQ,^3^^,^^8^^,^^10^^,^^12^, ^13^, ^14^, ^15^ but a few have reported return to work and return to normal activity outcomes.^3^^,^^30^^,^^31^ These results are important because they demonstrate a major goal of minimally invasive CTR techniques, like CTR-US, of reducing recovery time, thereby lessening the burden of work absenteeism. In the current study, 97% of patients returned to normal activity by 2 weeks and all patients had returned to work. These results with respect to return to work are faster than those reported for open CTR and similar to those reported for ECTR.^3^^,^^27^^,^^29^, ^30^, ^31^, ^32^, ^33^ Although these data are promising, we consider them preliminary because of the relatively small number of patients.

Detailed MRI analysis demonstrated morphological changes consistent with successful carpal tunnel decompression in all cases. A few studies have used ultrasound to evaluate morphological changes following CTR-US,^34^^,^^35^ but only Petrover et al^21^ has used MRI to quantify these changes. In that study, 129 patients who underwent CTR-US received baseline preoperative and postoperative MRI at 1 month. The results showed a complete transection of the TCL and nerve decompression in all cases. The metrics used to demonstrate nerve decompression were CSA of the median nerve and changes in nerve position, both measured at the level of the hamate.^21^ Results of our MRI analysis agree with the findings of Petrover et al,^11^ as both investigations reported a complete transection of the TCL in 100% of the cases, a significant increase in median nerve CSA, and a palmar shift of the median nerve. Our investigation expands this work by documenting increased carpal tunnel dimensions, reduced flattening ratios of the median nerve and carpal tunnel (ie, reduced flattening), and reduced median nerve T2 signal intensities. The results of these expanded metrics are all consistent with postoperative decompression following CTR-US and are consistent with previous data reported for morphological changes of carpal tunnel and its contents using MRI following endoscopic CTR and open CTR.^16^, ^17^, ^18^, ^19^, ^20^ Previous work has used signal intensity of the median nerve expressed as the ratio to the hypothenar muscles to avoid variations in signal encountered with surface coils.^17^^,^^19^ For the purpose of this investigation, T2 signal intensity of the median nerve was measured by placing a region of interest over the nerve at the level of the hamate and reporting as a single value. Results showed an 18% decrease in median nerve signal intensity after surgery. Whether this is a clinically significant value remains unclear without other comparable works with set a precedent. However, we hypothesize that this decrease is consistent with some degree of reduction in intraneural edema.^17^^,^^19^

Major strengths of this study are the use of validated patient-reported outcomes (BCTQ and *Quick*DASH), reporting return to normal activities and work status at 2 weeks, and the inclusion of morphological MRI data measured in blinded fashion for a subset of patients post CTR-US. The novel MRI data presented herein are consistent with carpal tunnel decompression and validate the ability of CTR-US to result in favorable morphological changes similar to those reported following open and ECTR.^16^, ^17^, ^18^, ^19^, ^20^ The primary limitations of this study include the retrospective review of prospectively collected data, the relatively small number of patients with 3–6-month clinical follow-up, and the inability to perform pre- and postoperative MRI scans on all patients. Although the clinical results in this study were favorable and commensurate with previously published intermediate-term outcomes for open and ECTR, additional research examining larger numbers of patients with longer follow-up will further define the role of CTR-US in the surgical treatment of patients with CTS.^10^^,^^29^^,^^33^ Although only 13 patients (17 wrists) were able or willing to have a postoperative MRI scan, the clinical outcomes of these patients were similar to those in the remainder of the group who did not have postoperative MRI scans, suggesting that the reported MRI results are representative of the entire sample of patients. Furthermore, the number of patients with pre- and postoperative MRI scans in the current study is commensurate with recently published studies using MRI to study morphological changes following CTR.^16^^,^^20^

In conclusion, CTR-US using WALANT in a procedure room setting was safe, effective, and resulted in morphological changes consistent with carpal tunnel decompression as demonstrated by MRI.
